# Resilience in the age of COVID-19

**DOI:** 10.1192/bja.2021.5

**Published:** 2021-02-17

**Authors:** Halley Kaye-Kauderer, Jordyn H. Feingold, Adriana Feder, Steven Southwick, Dennis Charney

**Affiliations:** 1 BA, is a fourth-year medical student at the Icahn School of Medicine at Mount Sinai, New York, New York, USA.; 2 MAPP, is a fourth-year medical student at the Icahn School of Medicine at Mount Sinai, New York, New York, USA.; 3 MD, is Associate Professor of Psychiatry and Associate Director for Research at the World Trade Center Mental Health Program at the Icahn School of Medicine at Mount Sinai, New York, New York, USA.; 4 MD, is Adjunct Professor of Psychiatry at the Icahn School of Medicine at Mount Sinai, New York, New York, and Professor Emeritus of Psychiatry at Yale School of Medicine, New Haven, Connecticut, USA.; 5 MD, is Anne and Joel Ehrenkranz Dean of the Icahn School of Medicine at Mount Sinai and President for Academic Affairs for the Mount Sinai Health System, New York, New York, USA.

**Keywords:** Trauma, trranscultural psychiatry, psychosocial interventions, resilience, neurobiology

## Abstract

Resilience is broadly defined as the ability to bounce back from adversity or trauma. Recent advances in resilience research have shifted away from merely describing individual characteristics towards focusing on the complex interactions between individuals and their dynamic personal, community and cultural contexts. It is clear that resilience involves both neurobiological and cultural processes. Neurobiological contributions include genes, epigenetics, stress-response systems, the immune system and neural circuitry. Culture helps to elucidate collective systems of belief and accepted positive adaptations. Importantly, resilience can also be affected by evidence-based interventions and deliberate practice on the part of the individual. This review seeks to understand resilience as a complex and active process that is shaped by neurobiological profiles, developmental experiences, cultural and temporal contexts, and practical training. It uses the COVID-19 pandemic as a case example to better understand individual and group responses to tragedy. We suggest practical recommendations to help populations around the world cope and recover from the global threat of COVID-19.

## LEARNING OBJECTIVES

After reading this article you will be able to:

• understand that resilience is complex, dynamic and context-dependent and involves the interactions between an individual and their changing environment
• understand that resilience is shaped by neurobiological profiles, developmental experiences, cultural conditions and practical training
• understand that COVID-19 can be used to better understand global resilience in the context of a universal stressor.

Stress and tragedy are inevitable parts of the human experience. From personal daily setbacks to shared trauma, individuals and communities learn to cope, survive and thrive in the face of adversity. Resilience is broadly defined as the ability to bounce back from adversity, serious threat or trauma (Southwick 2018; Feder 2019). Although resilient outcomes are much harder to achieve in the face of severe stress such as childhood abuse or chronic hardship such as living in poverty, even in the most dire situations, individuals may recover and find ‘relative resilience’ (Feder 2019).

**FIG 1:**
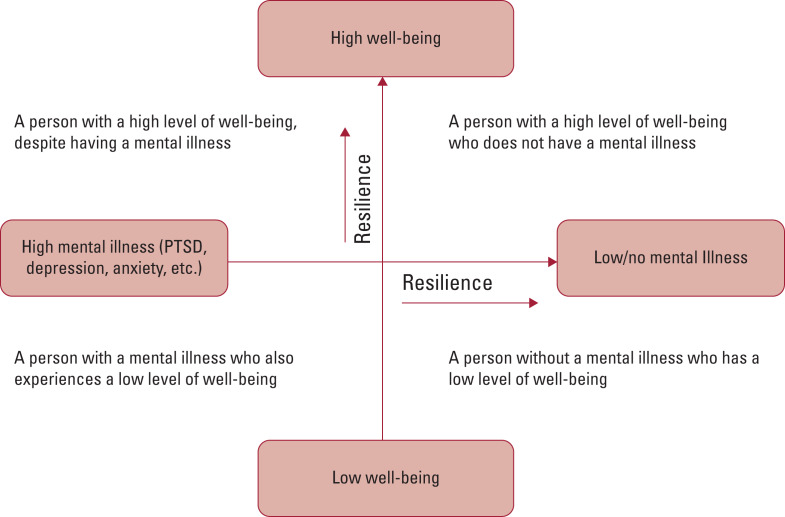
Well-being and mental illness as two related but distinct dimensions: resilience can move an individual along both dimensions towards greater well-being and lower mental illness (Westerhof 2010; MacKean 2011). PTSD, post-traumatic stress disorder. Figure adapted from MacKean (2011: Fig. 1), with permission.

Resilience operates both to combat the development of mental illness and to promote a state of thriving and well-being. It is important to recognise that well-being and mental illness comprise two related but distinct dimensions (e.g. the absence of a mental illness does not imply the presence of well-being and the presence of mental illness does not imply the absence of well-being: Fig. 1) (Westerhof 2010).

Throughout this article, we cite research that uses the development of psychopathology such as post-traumatic stress disorder (PTSD) as a proxy for a ‘less-resilient’ response to stress. However, this is an oversimplification. Resilience may also be viewed as continuing to function and grow even after the development of psychopathology. It is not the development of a disorder, but rather one's response to that disorder that reflects resilience.

In the past decade, studies of resilience have expanded beyond merely describing individual characteristics to focusing on the ‘complex choreography’ (Gottschalk 2020) between individuals and their changing personal and environmental contexts. Recent data have shown that resilience is best understood as a continuum with the potential to change across a lifespan based on biological, developmental and cultural determinants as well as evidence-based interventions and deliberate practice on the part of the individual.

Resilience has become even more relevant as our world faces a common stressor: the SARS-CoV-2 (COVID-19) pandemic. Studies investigating the psychological impact of the pandemic on healthcare professionals in China, Italy and the USA have shown significant symptomatology for generalised anxiety disorder, major depressive disorder and PTSD, especially among front-line workers (Lai 2020; Rossi 2020; Shechter 2020). Unsurprisingly, a pandemic of this scale may have lasting adverse outcomes for those most intimately involved. This crisis presents an unprecedented opportunity to draw on resilience research to study the connections between tragedy and resilience, struggle and accomplishment, and hardship and hope for our global community.

The present review posits resilience as the active and dynamic process of recovering from adversity. We begin by examining the baseline neurobiological and developmental patterns that influence one's ability to manifest resilient responses to stress. We then illustrate how personal resilience is shaped by cultural and environmental factors. We end with a discussion of interventions that promote resilience and implications for the COVID-19 pandemic. Box 1 defines some of the neurobiological terms used.

BOX 1 Neurobiological terms explained

**Neuropeptide Y (NPY)**: Associated with decreasing anxiety and hastening return to baseline after the nervous system reacts to stress.

**Haplotype**: A set of DNA variations or polymorphisms that tend to be inherited together. It can refer to a combination of alleles or to a set of single nucleotide polymorphisms (SNPs) found on the same chromosome.

**FK506 binding protein 5 (FKBP5)**: Involved in regulating the HPA axis and related to the rate at which cortisol levels return to baseline after a stressor.

**Hypothalamic–pituitary–adrenal axis (HPA axis)**: Major neuronal system that responds to stress with a complex set of reactions involving the hypothalamus, the pituitary gland and the adrenal glands.

**Genome-wide association studies (GWAS)**: An approach used in genetics research to associate particular variations in genes with specific diseases.

***SLC6A4***: Serotonin transporter gene that encodes for an integral membrane protein that transports serotonin from synaptic spaces into presynaptic neurons.

**Epigenetics**: The study of how a variety of internal and external environmental events can trigger biochemical reactions that regulate gene expression.

**Locus ceruleus–noradrenaline (LC–NA) system**: Major neuronal system implicated in arousal, attention and stress response. The locus ceruleus is the principal site for the brain's synthesis of noradrenaline.

**Noradrenaline**: Noradrenaline, also known as norepinephrine, is part of the sympathetic nervous system. It facilitates alerting and alarm reactions in the brain and is critical for responding to danger and for remembering emotional and fearful events.

**Corticotropin-releasing hormone (CRH)**: Peptide hormone released from the hypothalamus involved in the stress-response system and stimulation of the pituitary synthesis of adrenocorticotropic hormone (ACTH), as part of the HPA axis.

**Glutamate**: The most abundant excitatory neurotransmitter. Associated with stress vulnerability and resilience.

**Dopamine**: Associated with pleasurable feelings and plays a key role in the reward system of the brain. For this reason, it is an important factor in cravings and addictive behaviour.

**Brain-derived neurotrophic factor (BDNF)**: Acts to support the central and peripheral nervous system through the repair of existing neurons and growth of new ones.

**Endocannabinoid**: Endocannabinoid signalling is closely coupled with HPA axis signalling, modulating the stress response and facilitating recovery from stress.

**Oxytocin**: Associated with maternal behaviours, pair bonding, social communication, trust, social support and anxiety reduction.

**Cytokines (e.g. IL-6, CRP, TNF-α)**: Small proteins involved in cell signalling and communication. There are both pro-inflammatory cytokines and anti-inflammatory cytokines. Cytokines are involved in the activation of the HPA axis. Examples include interleukin 6 (IL-6), C-reactive protein (CRP) and tumour necrosis factor alpha (TNF-α).

## Neurobiology and stress

As a dynamic process, resilience involves complex interactions between genetic and environmental factors during development and throughout a person's lifetime. Here, we provide a non-exhaustive overview of the contribution of genes, epigenetics, stress-response systems, immune responses and neural circuitry function, with selected examples illustrating neurobiological mechanisms of resilience (Feder 2019).

### Genes

Genes influence the way individuals respond to stress. The heritability of PTSD, a psychiatric disorder exemplifying vulnerability to trauma, is known to be approximately 30–40% (Logue 2015). Additionally, studies have emphasised the importance of the stressor itself, both its nature and its severity, in dictating an individual's response to stress. More recently, twin studies have examined the heritability of resilience. Incorporating the number and severity of experienced stressors into statistical models yields a measure of ‘relative resilience’ for each individual – i.e. higher or lower resilience than predicted on the basis of characteristics of the sample as a whole. Using this approach, findings from a large twin study suggest that resilience is equally influenced by genes and environment (Amstadter 2014). Other twin studies have begun to examine heritability of trait resilience and of psychological characteristics known to be associated with resilience, for example prosocial attitudes.

Variation in a range of stress-response system genes, such as the alpha-2 adrenoreceptor or neuropeptide Y genes, has been implicated in differential responses to stress. For example, slower return to baseline of noradrenaline levels once a stressor subsides (Neumeister 2005) or lower production of neuropeptide Y in response to threat-related stimuli (Zhou 2008) result respectively in differential vulnerability or resilience to stressful events. In a key series of studies, variation in haplotypes of the *FKBP5* gene, coding for a chaperone protein that modulates hypothalamic–pituitary–adrenal (HPA) axis responsivity to stress, was shown to confer resilience or vulnerability to the development of trauma-related psychopathology in childhood (Watkins 2016). Paralleling the history of genetic research in psychiatric disorders, initial genetic studies of resilience focused on candidate genes. Examples include studies of protective alleles involved in modulating noradrenaline, amygdala and hippocampal responses to stress or threat (Zhou 2008) and others searching for protective alleles in more resilient survivors of childhood trauma (Cicchetti 2007).

Beyond confirming the importance of gene × environment interactions, studies suggest that some gene variants influence sensitivity to the surrounding environment, with broad implications for preventive interventions especially during childhood and adolescence (Belsky 2016). More recently, genome-wide association studies (GWAS) of resilience have emerged, facilitated by a growing number of publicly available data-sets and large research collaborations (Stein 2019).

### Epigenetics

Epigenetic studies also illuminate the impact of environmental factors on resilience. Certain environmental exposures throughout the lifespan, such as maternal care, social support and drug use, have been found to alter chromatin structure, thus affecting gene expression and an individual's susceptibility to trauma (Dudley 2011). In studies of adults, differences in methylation patterns of candidate genes, for example the serotonin transporter gene (*SLC6A4*) promoter, have been linked with differential psychological outcomes related to stressors of various degrees (Gottschalk 2020), as well as differential limbic system activity in response to sad and fearful stimuli (Ismaylova 2018).

Epigenetic changes during developmental years can have permanent effects on the brain. During prenatal and early postnatal life, the developing brain is particularly susceptible to environmental exposures via epigenetic modifications in the hippocampus resulting in long-lasting effects on stress response (Miguel 2019). During adolescence, exposures to excessive alcohol are associated with epigenetic changes in the frontal cortex, striatum and nucleus accumbens (Pascual 2009). Although much remains to be learned, studies have begun to elucidate the complex interactions between genotype, environment and epigenetics and their influence on resilient phenotypes.

### Stress-response systems

Resilience is heavily dependent on the efficient activation and termination of the stress response (Feder 2019), which is mediated by the HPA axis and the locus ceruleus–noradrenaline system and early life experiences. For example, increased stress in childhood (e.g. abuse or neglect) can lead to persistently elevated levels of corticotropin-releasing hormone (CRH), associated with decreased resilience persisting into adulthood (van Bodegom 2017). Chronic stress has also been linked to changes in glutamate-system function in adulthood, associated with synaptic atrophy in the hippocampus and prefrontal cortex (Deyama 2020). Of note, the hippocampus contains high levels of glucocorticoid receptors, which mediate negative HPA axis feedback, facilitating appropriate and efficient responses to stress. Additionally, the protective role of the dopaminergic system during responses to stress has been investigated, revealing that activation of dopaminergic neurons may facilitate resilience by dampening fear responses. The function of neuropeptide Y signalling is also potentially protective against stress and trauma. Higher levels of this anxiolytic neuropeptide may balance the actions of CRH in the brain, regulating fear responses (Sabban 2016). A range of other neuropeptide and neurotransmitter systems affect stress responses, including brain-derived neurotrophic factor (BDNF), endocannabinoid, oxytocin and other systems. Preclinical studies have identified novel molecular adaptations in more resilient animals, with translational potential to resilience in humans (Tan 2020). Taken together, these biological responses to stress will open new avenues for interventions to enhance resilience.

### The immune system

The immune system is another mediator of resilience. After a stressful event, glucocorticoids are released and inhibit the production of pro-inflammatory cytokines such as interleukin 6 (IL-6) (Gądek-Michalska 2013). In animal models, lower circulating levels of IL-6 prior to a stressful event have predicted subsequent resilience to stress (Gądek-Michalska 2013). In human studies, elevated systemic levels of IL-6 have been identified in individuals with treatment-resistant depression and PTSD (Hodes 2014), whereas dispositional positive affect, a characteristic of resilient individuals, has been associated with lower IL-6 levels (Stellar 2015). Studies have even linked positive affect with protection against developing infections such as the common cold and influenza after participants were deliberately infected with these viruses via nasal spray (Cohen 2006). Although these studies do not purport causality, they shed light on the potentially protective role of positive emotions and their impact on the immune system.

Elevated levels of C-reactive protein (CRP), a marker of peripheral inflammation, are also associated with the development of PTSD (Eraly 2014). Conversely, lower plasma levels of IL-6 and CRP, comparable to levels in trauma-unexposed controls, have been linked to recovery from PTSD (Gill 2013). Tumour necrosis factor alpha (TNF-α), another cytokine produced mainly by macrophages during acute inflammation, has also shown relevance. TNF-α has been associated with depression and TNF-α antagonists have shown promise for the treatment of depressive disorders, although there remains a lack of clarity on their adverse impact on the overall immune system (Brymer 2019).

Together, these studies highlight the complex and reciprocal interactions between the immune system and stress-response systems in mediating resilience and offer potential opportunities for novel treatment targets.

### Neural circuitry

Several studies aim to understand the neural underpinnings of resilience. Functional neuroimaging studies have examined neural circuitry involved in fear, emotion regulation, reward responses and cognitive flexibility, comprising interconnected regions of the amygdala, anterior cingulate cortex, hippocampus, ventral striatum and prefrontal cortex. Such research suggests that resilience is associated with lower activation of threat appraisal regions such as the amygdala, more efficient functioning of prefrontal areas subserving implicit emotion regulation and higher activation of dorsolateral prefrontal regions involved in cognitive control (Scult 2017; Chen 2018). Additionally, resilience seems to involve higher neural adaptability and more efficient use of neural–emotional resources for adaptive coping (Feder 2019). Of particular relevance is neuroplasticity, the brain's ability to respond to stimuli by reorganising its structure and connections. The discovery that connections between key brain regions can be strengthened or weakened following personal experiences helps contextualise resilience as dynamic over the lifespan.

## Transcultural resilience

Newer research has stressed the importance of the ecological perspective of resilience, which asserts the critical interplay between individuals, communities and cultures. Experts have argued that resilience must be understood as being context dependent, influenced by political, historical and temporal conditions.

Ungar (2008) has redefined resilience by placing culture at its core. He writes, ‘In the context of exposure to significant adversity, whether psychological, environmental, or both, resilience is both the capacity of individuals to navigate their way to health-sustaining resources, including opportunities to experience feelings of well-being, and a condition of the individual's family, community and culture to provide these health resources and experiences in culturally meaningful ways’ (Ungar 2008). Others have also contended that cultural values are the ‘bedrock’ of resilience as they provide information about suffering and adversity, positive adaptations and healthy functioning, and social and moral norms (Panter-Brick 2012). Culture offers guidelines for everyday living based on geographical identities, value systems and societal expectations (Theron 2015). It provides communities with collective belief systems and accepted strategies for coping. Importantly, an emphasis on culture helps to explain the enormous heterogeneity observed in responses to tragedy across the globe (Linz 2020).

Much of the foundational theory of resilience and culture is based on a Western notion of coping and strength (Ungar 2007; Theron 2015). Previously, there had been little investigation into non-Western cultures where conceptions of resilience, resources or politics differ. For example, in a collectivist society like Japan, studies show a greater emphasis on community support and national empowerment during recovery compared with the self-enhancing and personal strength-based approaches in the USA (Kaye-Kauderer 2019). Among Palestinian young adults, the concept of *samud* – ‘a determination to exist through being steadfast and rooted to the land’ – is at the heart of resilience (Nguyen-Gillham 2008).

Even within the same country or population, communities with different cultural ideals may react differently to disasters. Consider the findings from the Pathways to Resilience Study in South Africa, which investigated two distinct populations: one in a more traditional, collectivist and rural community and another in an individualistic, urban setting (Theron 2013). Resilient youth in the traditional society relied on supportive social networks and established ancestral values to cope and grow. Comparatively, those in the urban environment were resilient if they acted with fiscal and social independence. These examples emphasise the need for greater sensitivity in understanding how cultural contexts shape positive development following adversity.

To address this ambiguity, researchers have conducted large-scale multi-site studies to understand how culture shapes mechanisms of resilience. In a mixed-methods investigation, Ungar and colleagues worked across 14 sites in 11 countries to assess youths' responses to various tensions (Ungar 2007). They found that, although common aspects of resilience could be identified, youths from culturally distinct groups, namely Western and non-Western societies, identified unique patterns in their understanding and manifestation of resilience. Researchers identified 32 common domains of resilience related to (a) culture (spiritual or religious identification), (b) community (perceived social equity, safety, opportunities for work), (c) relationships (social competence, positive role models, perceived social support) and (d) the individual (optimism, problem-solving ability, insight), among others.

Future research should shed light on how individuals, communities and countries bounce back from disasters to ultimately differentiate the culturally distinct features of resilience from the more global, universal aspects. This will allow for the development of better measurement tools and the implementation of culturally appropriate interventions across diverse populations.

## Interventions to enhance resilience

Although there has been no universal approach to promoting resilience, efficacious interventions work by enhancing protective psychosocial factors and behaviours (Southwick 2018; Linz 2020). When targeting these protective factors, it is imperative to understand that interventions must be tailored to individuals’ developmental stages, their unique personal and cultural conditions, and the stressors themselves. It is also important to note that many interventions are designed and applied in Western models of mental illness and thus application outside of these tested cultures merits caution. Here, we present an overview of interventions used across age groups and cultures.

### Heterogeneity among resilience interventions

To date, ‘resiliency training programmes’ are a loosely defined set of interventions aimed to enhance resilience that lack standardisation in format, method of delivery, intervention and control groups, outcome measures and even the constructs used to define and measure resilience (Leppin 2014). A narrative review of 44 randomised control trials of resilience interventions for adults found that, despite methodological shortcomings, interventions showed small to moderate efficacy at enhancing resilience and mental health at 3-month follow-up (Leppin 2014; Linz 2020).

Among the studies analysed, interventions took place in groups, individual settings or a combination of formats. They occurred in-person, online, by phone or were multi-modal. Participants ranged widely and included teachers, students, soldiers, police officers, people with chronic physical or mental illnesses, traumatised populations and random samples from the general population. Interventions were compared with treatments, no treatment or waiting-list control groups. Sessions varied in length and frequency from a single 40 min session to several 120 min sessions. Programme content ranged across different therapeutic approaches (adaptation training, mindfulness, stress management, cognitive–behavioural techniques, etc.), and some programmes focused on the explicit teaching of phenotypic resilience features such as emotion regulation, optimism or self-efficacy.

Although this heterogeneity has garnered criticism for potentially diluting the field of resilience intervention research (Leppin 2014), it is important to acknowledge that different aspects of resilience may be differentially valued by different populations; there may be no single ‘one-size-fits-all’ intervention for building resilience.

### Child and adolescent interventions

Individuals and groups at different developmental stages warrant distinct age-appropriate and context-dependent interventions. For example, young children exposed to adversity are particularly susceptible to developing mental and physical disorders as a result of the vast neural growth and plasticity during this period (Shonkoff 2012). This plasticity also makes them ideal candidates for focused interventions. Early effective intervention programmes may be caregiver and family based, school based or community based. Long-term school-based programmes for children in Bhutan, Mexico and Peru have shown an increase in well-being and academic performance after students underwent a 15-month intervention that focused on, among other skills, the quality of interpersonal relationships, empathy and altruism, mindfulness, effective communication and emotion management (Adler 2016).

### Adult interventions

Interventions for adults typically draw on advanced processes of cognition and emotion processing. They range from pre-trauma training and preventive approaches to early post-trauma and long-term programmes.

### Prevention

Preventive programmes for mental health may target specific skills, such as cognitive reframing and support-seeking, hardiness training, or relaxation and mindfulness guidance (Horn 2018). Perhaps the most widely used examples are those employed by the US army: master resilience training (MRT) and pre-deployment stress inoculation training (PRIEST). These courses teach soldiers stress control, coping skills, support-seeking and cognitive reappraisal (Griffith 2013; Hourani 2016). Soldiers who have completed MRT have reported enhanced coping and fewer symptoms of poor behavioural health during subsequent stressful times (Griffith 2013). Additionally, there is evidence that soldiers without baseline mental health problems who complete PRIEST are protected against developing symptoms of PTSD (Hourani 2016). Given the inevitability of exposure to traumatic situations among soldiers, the case for preventive resilience interventions is obvious. With increasing global stress, preventive programmes for general populations might be of great utility to minimise adverse mental outcomes.

### Early post-trauma and short-term interventions

Early post-trauma and short-term resilience interventions focus on exposure therapy and the development of effective coping mechanisms. In one study, individuals who suffered from DSM-IV criterion A trauma who engaged in a modified exposure therapy programme in the emergency department hours after experiencing the trauma reported lower symptoms of PTSD and depression 3 months later compared with the control group (Rothbaum 2012). In a similar study, survivors of sexual assault who were shown a short video teaching coping strategies within 72 h of their assault also reported lower PTSD and depressive symptoms up to 6 months later (Resnick 2007). Although these results are promising, further research is warranted to characterise the strengths and pitfalls of these early post-trauma interventions.

### Long-term interventions

Long-term resilience training programmes vary greatly, but most aim to promote the development of lasting psychosocial, behavioural and cognitive tools (e.g. social support, optimism, physical exercise, emotion regulation and cognitive reappraisal). One intervention for US combat veterans with PTSD and their partners uses a couples-based treatment called strategic approach therapy (SAT). This therapy targets avoidance symptoms of PTSD by encouraging effective communication, intimacy and anxiety reduction (Sautter 2009).

### Pharmacological interventions

Pharmacological interventions may be employed in addition to psychosocial approaches or as stand-alone treatments to alter fear responses, emotion regulation, memory and attention and to enhance prosocial behaviours (Kelmendi 2016). For example, several drugs have been proposed to increase resilience by regulating the sympathetic nervous system (e.g. neuropeptide Y) and HPA axis (e.g. CRH antagonists, dehydroepiandrosterone), improving neurogenesis, preventing neuronal damage (e.g. antidepressants) and controlling memory formation for traumatic events (e.g. beta blockers). The combination of medication and psychotherapy has been shown to be superior to either type of treatment alone (Kamenov 2017).

### Summary

We have highlighted the great deal of heterogeneity among resilience training programmes. Given the discussion of various cultural conceptions of resilience, this diversity is necessary but also limits our ability to make valid comparisons across interventions. Importantly, the goal for many of these programmes is not just to mitigate the risks of developing negative psychopathology, but also to teach skills that promote mental health and sustainable well-being. Although many distinct interventions aim to accomplish this, the common thread among them is an emphasis on behavioural change and deliberate practice. Akin to working a muscle, the cultivation and development of resilience takes time, practice and, often, support from others in order to see positive changes in phenotype and underlying physiology.

## The COVID-19 pandemic and future directions for resilience research

### What else affects resilience?

As discussed, individual responses to stress and adversity are mediated by neurobiological, psychosocial and cultural factors. Although many will recover or endure a brief symptomatic period, some may suffer lasting psychological consequences. The idea that resilience is context dependent means acknowledging the profound impact of past traumatic experiences, pre-existing psychological and medical conditions, and access to resources. In other words, exhibiting and building resilience is easier for some than for others. This notion is particularly salient when examining the diversity of responses to the COVID-19 pandemic.

Studies repeatedly show that the frequency and intensity of stress matter (Hobfoll 2011) and those who have experienced childhood trauma or chronic stress are less likely to manifest resilience (Alim 2008). Individuals with pre-existing psychiatric conditions such as major depressive disorder will also need to work harder to adapt, owing to overwhelming feelings of hopelessness or anhedonia that may prevent their active practice of resilience. In a similar vein, chronic medical conditions or severe injuries may make it more difficult for individuals to employ cognitive and physical coping strategies. The severity of COVID-19-related illness can similarly affect survivors’ resilience.

Finally, an individual's resilience is strongly influenced by external support and available resources. Consider for a moment the effects of COVID-19 for an individual with a stable income, housing and established support systems compared with an uninsured individual who lost their job and lives in a small and crowded multi-generational apartment. Clearly, varying levels of wealth, job security, social support and socioeconomic status make it easier for some to overcome the adversity caused by COVID-19. Early data have shown that individuals and communities most affected are those with the least access to healthcare, job security, community services and other basic resources (Ahmed 2020). In the USA, communities of colour, including Black, Latinx and Native American populations, are disproportionally affected owing to the aforementioned factors as well as systemic racism within the healthcare system and society (Fortuna 2020). Resources must be allocated to ensure that these suffering communities are given the greatest chance to survive and thrive. COVID-19 presents an opportunity to create actionable changes that lead to the establishment of a stable ground for all to stand upon.

**FIG 2:**
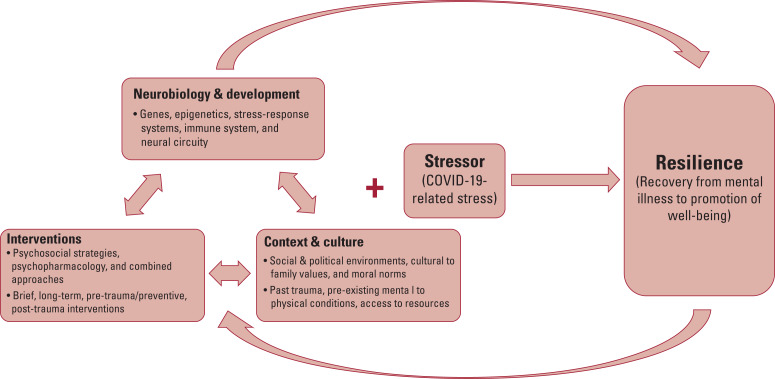
An integrated model of resilience. Neurobiology and developmental profiles interact with contributions from context and culture as well as progress made from evidence-based interventions. At the core of resilience is the interaction between these three systems and the stressor itself. In this article, we use the COVID-19 pandemic as an example of a universal stressor that may have an impact on diverse populations and cultures around the world in unique and shared ways. Importantly, this relationship moves in both directions as the resilience developed and manifested by individuals can also feed back to further influence their neurobiology, cultural context and practised interventions.

Another mediating factor that predicts how an individual or community will recover from stress is the stressor itself. The world now faces a universal stressor: COVID-19. Although the pandemic has disproportionate impact depending on racial or ethnic minority group status, geography and personal proximity to the virus, the universal nature of this tragedy may allow for tighter control of a crucial variable (the stressor) in calculating the complex equation that defines resilience (Fig. 2). This presents a unique opportunity for several applications of future research. For example, researchers across the world might choose to observe global responses to further validate personal/individual, social and cultural determinants of resilience. It also poses an opportunity to better understand and eventually predict the trajectories of individuals and groups with distinct neurobiological, developmental, psychosocial and cultural profiles. This research might involve international surveys, intervention programmes, coordinated qualitative interviews, or other forms. Despite variation, this work should strive for a broad, dynamic and global approach.

### Recommendations and interventions

As the pandemic progresses, we can draw on findings presented in this review to support the development, scaling and implementation of resilience interventions. Examples are summarised in Box 2.

BOX 2 Recommendations for the COVID-19 pandemic

**Opportunities to study resilience amid the pandemic**

• Studying the ‘inequality of resilience’, including past traumatic experience, pre-existing psychological and medical conditions and access to resources
• Consolidating global efforts to study resilience in the context of a universal stressor
• Detailing personal, social and cultural determinants of resilience
• Understanding the trajectories of individuals and groups with distinct neurobiological, psychosocial and cultural profiles

**Capitalising on resilience factors amid the pandemic**

• Positive affect: holding positive emotions (joy, love, gratitude, etc.) alongside negative emotions (fear, concern)
• Cognitive reappraisal: seeing this time as an opportunity to re-examine societal institutions, reframing quarantine as an opportunity to reconnect with family members and others and improving on a hobby or interest
• Social support: maintaining social connection through virtual methods and volunteering to help those who are older and isolated
• Spirituality: seeking meaning, purpose and direction in life from spiritual connections such as religious practice, rituals and/or non-religious spiritual forms such as nature or music

**Community-based recommendations amid the pandemic**

• Screening and seeking out high-risk individuals within communities
• Tailoring interventions to specific populations and cultures and embracing a wide range of interventions from both Eastern and Western traditions
• Proactively linking individuals to appropriate resources, including access to basic needs
• Heeding this opportunity to de-stigmatise and normalise the pursuit of mental healthcare on a global scale
• Coordinating efforts between local and national governments, healthcare systems, educational institutions and employers

#### Positive affect

Positive emotions have been shown in the laboratory to mitigate the negative impact of stress by supporting more efficient cardiovascular recovery and lower autonomic arousal (Tugade 2004). As previously discussed, positive affect is also associated with lower susceptibility to developing infections (Cohen 2006). Accordingly, there may be a protective role for positive affect during the pandemic to support not only psychological well-being, but also physical health and protectionfrom the virus.

Studies show that resilience may be mediated by the ability to hold positive emotions alongside negative emotions during times of stress. Classically, this dialectical coding of emotions has been attributed to East Asian cultures, which are more likely to seek a balance between positive and negative emotions compared with, for example, North Americans, who tend to avoid this middle ground (Miyamoto 2011). In the aftermath of the terrorist attacks of 11 September 2001 (9/11) in the USA, however, American college students with the most resilient responses were those who maintained gratitude and hope in the midst of the slurry of negative emotions (Fredrickson 2003). Although this dialectical thinking may be more culturally concordant with Eastern traditions, the observation of this phenomenon in a Western sample reveals its potential as a universal resilience factor.

Individuals and communities may practise resilience by seeking gratitude for what has not been lost, embracing love, savouring moments of joy and effortfully maintaining positive emotions. It is imperative during these times to maintain a positive, yet realistic, understanding of the evolving situation and this should be encouraged by role models and leaders throughout the world.

#### Cognitive reappraisal

A habit of cognitive reappraisal, or the deliberate use of thoughts to reframe negative circumstances, can lead to decreased physiological arousal and anger in adults (Gross 2002). Importantly, reappraisal involves an operational decision that must be frequently reaffirmed with a desire to achieve personal growth (Yehuda 2007; Southwick 2018). This process has been shown to activate the prefrontal cortex and inhibit the amygdala, dampening negative emotional responses (Ochsner 2012). Thus, by actively reappraising aspects of the pandemic, individuals may be better able to control their emotional reactions to it. For example, communities around the world are already taking the pandemic as an opportunity to re-examine societal institutions, from work environments to healthcare and education systems to gender equality. Individuals may conceptualise quarantine as a chance to (re)connect with family, cultivate a hobby or feel more united with others in their shared stress. Additionally, as the pandemic evolves, it is to be hoped that countries across the world will apply present learning to the development of robust and agile strategies for managing future pandemic responses.

#### Social support

Maintaining social connection amid the pandemic is essential, especially in the context of ‘social’ (physical) distancing recommendations. Social connections are strongly associated with improved psychological outcomes and lower rates of PTSD after severe trauma, which may be explained by a number of biological phenomena, including reduced fear responses, activation of reward circuitry, oxytocin release and decreased cortisol levels (Tsai 2012). Robust links have been observed between strong social ties, good mental health, good physical health and longevity, as well as reduced rates of psychopathology following traumatic stress. Additionally, social isolation and loneliness have been associated with cardiovascular, immune and mental health problems (Holt-Lunstad 2010).

The pandemic creates an enormous challenge to remaining socially connected. Although social distancing is crucial to slow viral spread, it may come with significant costs when not supplanted with other forms of connection (e.g. virtual means). These costs may be particularly high for older adults, who are at increased risk for the toxic effects of the virus and may have less access to and comfort with virtual communication. Interventions aimed at enhancing access to technology and augmenting safe methods of social connectedness for elderly populations may be of great utility to both older adults and those caring for them (theoretically younger, healthy individuals). Individuals who volunteer to support older adults may themselves experience an enhanced sense of meaning and altruism, other facets of resilience. Synergising opportunities to meet the needs of various populations may enhance community resilience across generations.

#### Spirituality and religion

Spirituality – which may be defined as the attempt to seek meaning, purpose and direction in life from a higher power, universal spirit or God – is another potent resilience factor to help individuals recover from disaster (Meichenbaum 2008). Whether through a particular religious practice, a broader belief in something larger or a feeling of connection with all life forms, many individuals turn to spirituality in times of hardship. Following the 9/11 terrorist attacks, a national survey found that 90% of Americans turned to spiritual activities to cope (Schuster 2001). Additionally, religion appears to be especially significant for populations facing trauma in low- and middle-income countries where religion holds immense cultural significance (Ali 2012), as well as for those in the most unpredictable situations with the fewest resources (Hill 2003).

Spirituality or religious practice may provide a pathway to resilience by helping individuals find more benevolent meaning and community support amid unexplainable situations. In this way, spirituality, cognitive reframing and social support go hand in hand.

Individuals across the globe may rely on spiritual practices throughout the pandemic, including engagement with prayer or scripture, ‘attending’ religious services from home via the internet, turning to clergy or religious leaders for guidance or performing spiritual acts. Non-religious individuals may turn to other forms of spiritual engagement, including interacting with nature, participating in community service, engaging in meditation or drawing on creative art processes.

### Final thoughts

Although the tendency to engage in resilient behaviours may be easier for some than others, practising such strategies over time can eventually modify neurobiology to reinforce resilient behaviour via neuroplasticity. Importantly, practices should be tailored to specific populations and cultures. In one recent study looking at the psychological impact of COVID-19 on the general public in China, the authors suggest several psychological interventions (Wang 2020), including globally shared modalities such as cognitive–behavioural therapy (CBT), along with traditional Chinese medicine such as self-administered acupressure and emotional freedom techniques. In another article, researchers from the USA present multiple coping strategies based in both Western and Eastern traditions, including behavioural activation, acceptance-based coping, mindfulness practices and loving-kindness meditation to decrease stress and promote resilience (Polizzi 2020). As more research emerges, coordinated global efforts are needed to track the effectiveness of these interventions across diverse populations.

Since avoidance is a common response to trauma and there is a high degree of stigma in many communities associated with seeking help for psychological problems, interventions should be made even more public and widely accessible. For example, in settings such as hospitals, where exposure to the pandemic is inevitable, interventions might be offered to employees with a default opt-out rather than opt-in option, to change norms. Additionally, in communities hit hardest, screening for trauma should be augmented (e.g. by primary care physicians, community healthcare workers, teachers, employers and others) with opportunities to connect those who screen positive to resources. We have before us an opportunity to destigmatise and normalise the pursuit of mental healthcare on a global scale. Doing so will require cooperation and resources from local and national governments, healthcare systems, educational institutions, employers and others to ultimately advance the mental health and resilience of our global population.

## Supplementary material

For supplementary material accompanying this paper visit https://doi.org/10.1192/bja.2021.5. click here to view supplementary material

## MCQs

Select the single best option for each question stem

1 Increased resilience has the ability to move an individual towards:
  a high well-being and low mental illness
  b high well-being and high mental illness
  c low well-being and high mental illness
  d low well-being and low mental illness
  e none of the above.
2 Genes found to be implicated in differential responses to stress include:
  a alpha-2 adrenoreceptor
  b neuropeptide Y (*NPY*)
  c FKBP5
  d SLC6A4
  e all of the above.
3 Resilience interventions are defined in this article as:
  a a set of standard and chosen interventions that are fixed for all populations
  b a set of interventions based only on changing maladaptive thought patterns
  c a set of interventions based only on changing maladaptive behaviours
  d a set of interventions that inherently lack standardisation in nearly every domain
  e none of the above.
4 This article describes an equation that loosely defines resilience in terms of:
  a neurobiological and developmental factors, culture and environmental contexts, interventions and the stressor
  b neurobiological factors, social support systems, mental illness and the stressor
  c cultural contexts, available resources, education and the stressor
  d cultural contexts, interventions, job security and the stressor
  e personal attributes, family history, cultural contexts and the stressor.
5 Recommendations for recovering from the psychological impact of COVID-19 presented in this article include drawing on factors such as:
  a role models, social support, optimism and moral compass
  b positive affect, cognitive reappraisal, social support and spirituality
  c facing fears, physical exercise, spirituality and social support
  d meaning and purpose, positive affect, physical exercise and role models
  e social support, cognitive reappraisal, moral compass and physical exercise.

MCQ answers 1 a 2 e 3 d 4 a 5 b
